# Combining CEUS and ultrasound parameters in thyroid nodule and cancer diagnosis: a TIRADS-based evaluation

**DOI:** 10.3389/fendo.2024.1417449

**Published:** 2024-06-17

**Authors:** Andreea Borlea, Luciana Moisa-Luca, Alina Popescu, Felix Bende, Dana Stoian

**Affiliations:** ^1^ Division of Endocrinology, Department of Internal Medicine II, “Victor Babes” University of Medicine and Pharmacy, Timisoara, Romania; ^2^ Centre for Molecular Research in Nephrology and Vascular Disease, “Victor Babeş” University of Medicine and Pharmacy, Timişoara, Romania; ^3^ Division of Gastroenterology and Hepatology, Department of Internal Medicine II, “Victor Babes” University of Medicine and Pharmacy, Timisoara, Romania

**Keywords:** TIRADS, thyroid cancer, contrast-enhanced ultrasound, thyroid CEUS, microvascular

## Abstract

Contrast-enhanced ultrasonography (CEUS) has been established as a diagnostic tool for assessing microvascularization, essential for understanding angiogenesis in neoplastic development. Aim: This study assesses the effectiveness of CEUS as a supplementary tool to TIRADS in enhancing the ultrasound-based diagnosis of thyroid cancer. Methods and Materials: Over one year, 157 nodules in 133 patients, with predominantly solid thyroid nodules, were examined using ultrasound and CEUS and underwent thyroidectomy, allowing for a comparison of ultrasound findings with pathological reports. Results: Thyroid cancer was identified in 31.21% (49/157) of cases. Significant CEUS high-risk features included inhomogeneous enhancement, enhancement defects, and complete hypoenhancement (AUC 0.818, 0.767, 0.864 respectively). Nodules exhibiting any of these features were classified as high-risk in CEUS. The diagnostic performance of TIRADS improved when combined with CEUS, with AUC increasing from 0.707 to 0.840 and improved sensitivity. Conclusion: The integration of CEUS with TIRADS significantly enhances the diagnostic accuracy and specificity in identifying thyroid cancer. This combination proves to be a more effective method for risk stratification and diagnosis, highlighting the value of CEUS as an adjunctive tool in thyroid cancer evaluation.

## 1 Introduction

Thyroid nodules are prevalent, with up to 65% of the general population having one or more identified thyroid nodules (1–3). Today’s thyroid nodule clinicians are faced with a new task: avoiding over-diagnosing low-risk malignancies, without compromising the likelihood of discovering rare advanced or higher-risk tumors that need immediate treatment (4). Overdiagnosis defines discovering conditions that will never cause illness or death, where risks exceed benefits (5).

The first step in evaluating a thyroid nodule, whether it is detected during a clinical examination or incidentally, involves performing a cervical ultrasound (US) evaluation and assessing the patient’s clinical risk factors (5–9). Based on the outcomes, additional diagnostic tools like fine-needle aspiration thyroid cytology and molecular testing may be necessary for a limited number of thyroid lesions (10–12). The diagnostic performance of current Thyroid Imaging Reporting and Data System (TIRADS) can be enhanced even further by integrating contrast-enhanced US (CEUS) with the classic features.

CEUS has the ability to serve both as a diagnostic tool and as a means of carrying out procedures in cases of nodular thyroid pathology, such as to provide guidance during ablation procedures on thyroid nodules and metastatic lymph nodes (13, 14). Additionally, it can be employed to assess the outcomes of thyroid surgery, to determine the recurrence of disease and monitor the aspects of remnant lymph nodes following surgery (5, 15). As a diagnostic tool, contrast-enhanced ultrasonography (CEUS) is a recognized method for assessing microvascularization, which is crucial as angiogenesis underlies neoplastic development (16). The CEUS enhancement patterns of thyroid nodules compared to the surrounding parenchyma are used to diagnose them qualitatively and quantitatively (17). Hypoenhancement observed in CEUS is a major pattern indicative of malignancy in thyroid nodules. It occurs when the growth of the tumor surpasses the development of new blood vessels, resulting in reduced enhancement. This can be attributed to factors such as necrosis and the creation of emboli inside the tumor (17, 18). As an equivalent quantitative measure, the nodule-to-perinodule peak intensity ratio demonstrated the best diagnostic efficacy for a cutoff value of 0.9 (18). Malignant thyroid nodules often exhibit a heterogeneous enhancement, explained by intranodular fibrosis, calcifications and areas of necrosis (19, 20). A quantified value of heterogeneity has been determined using Adobe Photoshop as standard deviation/mean intensity×100, which was considerably greater in malignant nodules (20).

This study aims to rigorously evaluate the efficacy of integrating thyroid CEUS with the established TIRADS framework. As a first step, it seeks to detect potential malignancy features in qualitative CEUS assessment and to define CEUS high-risk. The main goal is to explore whether the addition of CEUS can significantly refine the process of ultrasound-based risk stratification and diagnosis in thyroid nodules, potentially leading to more informed clinical decision-making and better patient outcomes.

## 2 Materials and methods

### 2.1 Patients

**Figure 1:**
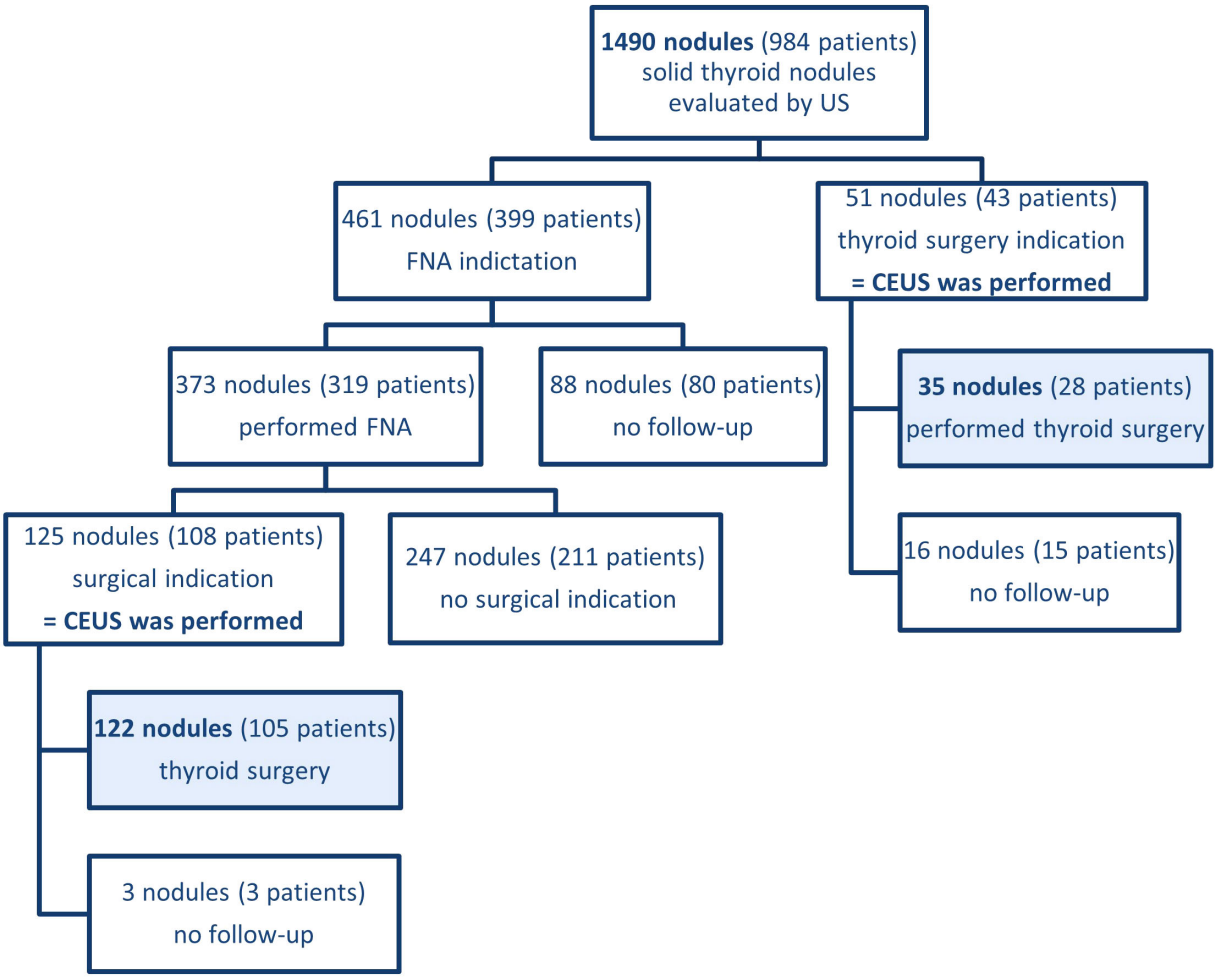
Patients included in our study from all evaluated thyroid nodules in our institution in the inclusion period. US, ultrasound; CEUS, contrast-enhanced ultrasound; FNA, fine-needle aspiration.

In this prospective study, we reviewed the medical records of 984 patients with 1490 thyroid nodules (**Figure 1**), evaluated by thyroid ultrasound at our institution from November 2022 to January 2024. 157 nodules in 133 patients met the following inclusion criteria: (a) they had ultrasound-diagnosed thyroid nodules; (b) they underwent both conventional ultrasound and CEUS examinations; (c) the size of the nodules were between 5–30 mm in maximum diameter; (d) the nodule composition was mostly solid; (e) they underwent surgery with pathological results available for diagnosis golden standard. Exclusion criteria were: (a) cystic or predominantly cystic thyroid nodules; (b) profound nodules located too deeply that cannot be evaluated properly by means of ultrasound; (c) other causes of incomplete imaging data; (d) hyperfunctional nodules and (e) absence of pathology reports at the moment of inclusion. CEUS was performed in nodules EU-TIRADS score 3–5 with thyroid surgery indication. FNA was indicated in EU-TIRADS score 3 nodules larger than 20 mm, in EU-TIRADS 4 nodules bigger than 15 mm and in EU-TIRADS 5 nodules bigger than 5–10 mm, taking individual risk factors into consideration as well. Surgery was indicated in patients with cytology results covering Bethesda categories IV, V and VI, in benign cytology (Bethesda II) with compressive symptoms; in Bethesda III FNA was either repeated if EU-TIRADS score was 4 or 5 and 3-month US follow-up was indicated in EU TIRADS score 3. This study received approval from the local ethics committee (approval number 235/2021), and all patients provided written informed consent.

### 2.2 Ultrasound and CEUS evaluation

In all patients, conventional US evaluation was performed by the same examiner, with 10 years of expertise in thyroid US (A.B.). The process began with positioning the patient supine with a pillow under their neck to aid hyperextension, with a generous amount of coupling gel applied between the US probe and the skin. All nodules were described according to the European TIRADS (7). In this regard, the size, shape, margins, echogenicity, composition, and presence of echogenic foci were described in each lesion and the TIRADS category was established. The examination utilized the SuperSonic MACH^®^30 ultrasound device (Supersonic Imagine, Aix-en-Provence, France) along with UltraFast™ image acquisition software and a high-frequency linear probe (5–18 MHz).

Following the standard US assessment, the same experienced examiner conducted the thyroid CEUS examination using the same US equipment. A low mechanical index was set automatically by the US system (<0.1) and the operator prospectively recorded the ultrasound frame for 3 minutes, with the probe held still, the nodule in the center of the field of view, visualized entirely, and with some neighboring thyroid parenchyma in the field of view, avoiding probe movement and asking the patient to breathe superficially and avoid swallowing. An injection of 1.6 ml SonoVue (Bracco, Italy) contrast agent was administered in a peripheral vein, for each nodule analyzed, followed by a 10 ml saline flush. In developing the CEUS scale for evaluating thyroid nodules, careful consideration was given to the selection of imaging features. The following qualitative parameters were assessed by reviewing the acquired images post-examination: (a) the enhancement degree - hyopenhancement, isoenhancement or hyperenhancement), compared with adjacent thyroid parenchyma, (b) the homogeneity of enhancement - homogeneous or inhomogeneous, (c) the presence or absence of a complete, hyperenhanced peripheral ring, (d) intranodular enhancement defects, characterized by areas of absent enhancement inside the nodule and (e) the washout time - early or similar to the neighboring thyroid tissue (21–25). Aiming at enhancing the objectivity and reliability of the diagnostic scale, we opted to exclude the wash-in pattern from our assessment criteria. Although some studies categorize the wash-in pattern as centripetal, centrifugal, or non-concentric, our team found this feature to be highly subjective with significant inter-operator variability. Furthermore, the literature reflects inconsistent performance of this characteristic in diagnostic accuracy.

### 2.3 Upgrading TIRADS category with CEUS

**Figure 2:**
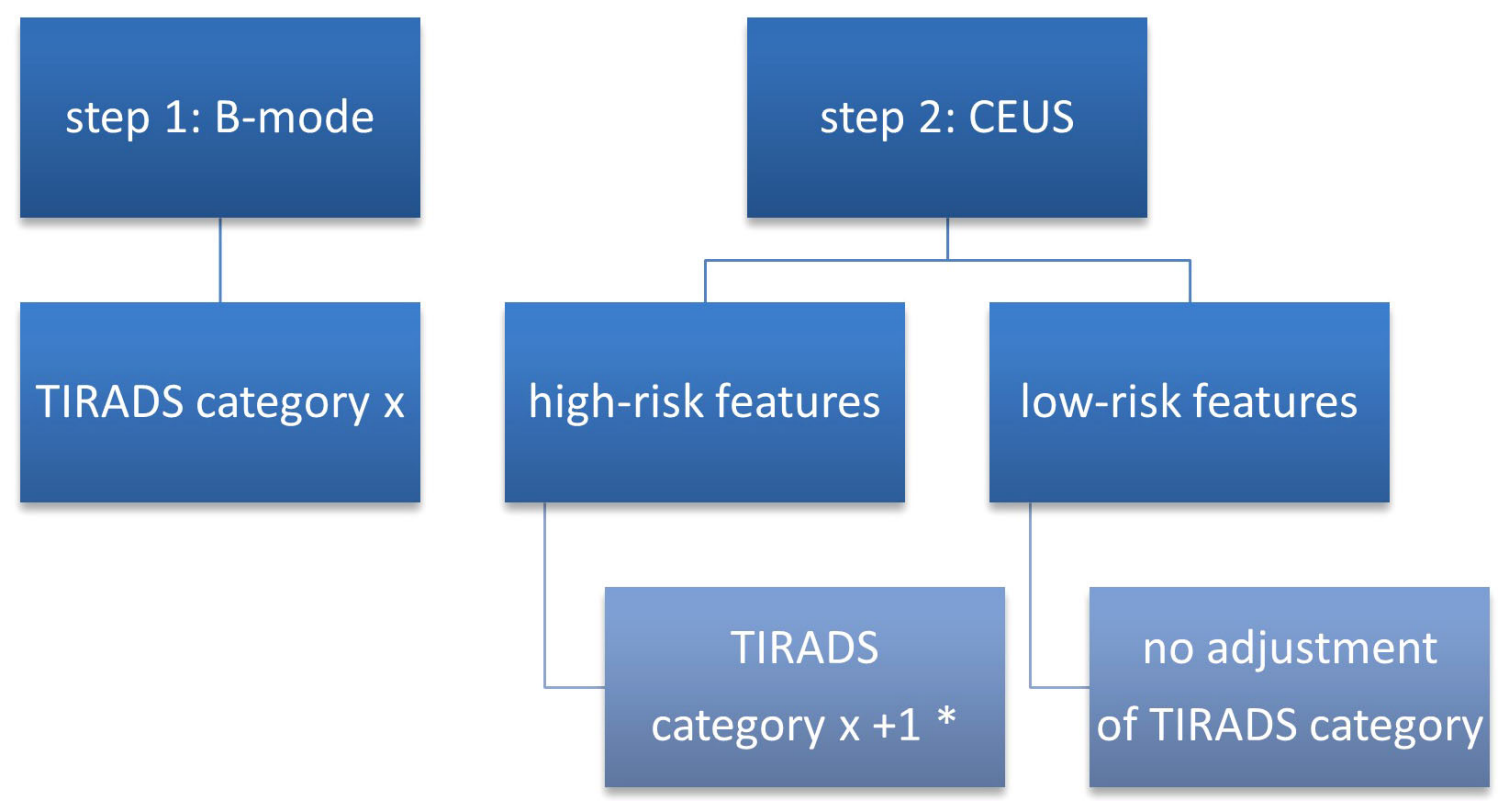
TIRADS + CEUS upgrade algorithm. CEUS, contrast-enhanced ultrasound; TIRADS, Thyroid Imaging Reporting and Data System; * - except from category 5 which remains unmodified.

After the initial attribution of each nodule to a TIRADS category, a risk-upgrade was made in all cases which presented high-risk features in CEUS (**Figure 2**). Considering that current CEUS data for thyroid nodules are not validated by the guidelines, we did not perform a downgrade of risk in nodules with high-risk features in B-mode which presented low-risk features in CEUS.

### 2.4 Statistical analysis

The statistical analysis was conducted using MedCalc V19.4, provided by MedCalc Software Ltd. in Flanders, Belgium. Descriptive statistics were applied to demographic data and ultrasound findings. The distribution of the numerical data was assessed using the Kolmogorov-Smirnov test. For normally distributed numerical variables, the mean and standard deviation were reported, while the median and interquartile ranges (25–75%) described those with non-normal distributions. Qualitative variables were presented using figures and percentages. The Mann-Whitney U-test was utilized for non-parametric variable analysis, while the t-test was used for evaluating parametric variables. In order to evaluate the diagnostic precision of TIRADS, CEUS and TIRADS+CEUS in identifying thyroid cancer, receiver operating characteristic (ROC) curves were employed. An optimal threshold value was determined to differentiate between benign and malignant nodules for these diagnostic algorithms. Interval likelihood ratios were calculated for the multiple ultrasound-based scores and algorithms. Furthermore, post-test probabilities were computed and compared for the different diagnostic approaches. A p-value of 0.05 or less was considered as indicative of statistical significance.

## 3 Results

### 3.1 Thyroid nodules diagnosis

Out of the 133 patients (157 nodules) analyzed, median age 48 years, mostly women (90.5%). Cancer was detected in 49 cases (43 papillary, 5 follicular and 1 medullary thyroid cancer). Nodule size, gender, and age did not significantly differ between cancers and benign nodules. However, in terms of TIRADS category, median score assigned was 3 for benign and 4 for the malignancies, the difference presenting statistical significance. The general characteristics of the patient and ultrasound diagnosis (TIRADS) in benign and malignant pathology are described in **Table 1**.

**Table 1:** Patient and ultrasound characteristics in benign and malignant thyroid nodules. .

	All nodules (n=157)	Benign nodules (n=108)	Malignant nodules (n=49)	p-value
Patient age	48 (34–55)	47 (31–55)	53 (44–55)	0.120
Gender	90.5%	88.8%	93.8%	0.489
Nodule size	2.1 (1.5–2.4)	2.1 (1.5–2.4)	2 (1.4–2.4)	0.790
EU-TIRADS category	3 (3–4)	3 (3–4)	4 (3–5)	<0.0001

EU-TIRADS, European Thyroid Association Thyroid Imaging Reporting and Data System.

The diagnostic accuracy of the B-mode evaluation, as determined through TIRADS, was found to be moderate, with an AUC of 0.707.

### 3.2 CEUS characteristics

There were statistically significant differences between cancer and benign lesions for all CEUS parameters included in our analysis (**Table 2**).

**Table 2:** The prevalence of each CEUS qualitative feature in malignant and benign thyroid nodules analyzed in our study.

CEUS feature		BENIGN (n=108)	MALIGNANT (n=49)	p
enhancement degree	hypoenhancement	15 (13.8%)	26 (53.1%)	<0.0001
	iso- or hyperenhancement	93 (86.2%)	23 (46.9%)	
homogeneity of enhancement	homogeneous	26 (24.1%)	6 (12.2%)	<0.0001
	inhomogeneous	82 (75.9%)	43 (87.8%)	
complete, hyperenhanced peripheral ring	present	81 (75%)	1 (2%)	<0.0001
	absent	27 (25%)	48 (98%)	
enhancement defects	present	4 (3.7%)	28 (57.1%)	<0.0001
	absent	104 (96.3%)	21 (42.9%)	
washout time	early	22 (20.4%)	23 (46.9%)	0.0008
	late-phase persistence	86 (79.6%)	26 (53.1%)	

However, in terms of diagnostic accuracy, when each parameter was analyzed individually, the best performance was detected for inhomogeneous enhancement as predictive for cancer and the presence of a complete, peripheral ring as predictive for benignity in thyroid nodules (Area under the ROC curve - AUC >0.8). The presence of enhancement defects inside the nodule had the highest specificity for detecting thyroid cancer, followed by hypoenhancement. **Table 3** presents all diagnostic parameters for CEUS parameters.

**Table 3:** Diagnostic performance of CEUS parameters.

	AUC	Se	Sp	PPV	NPV
hypoenhancement	0.695	53.1%	86.1%	63.4%	80.2%
inhomogeneous enhancement	0.818	87.8%	75.9%	62.3%	68.8%
complete, hyperenhanced peripheral ring	0.864	98%	75%	64%	98.8%
enhancement defects	0.767	57.1%	96.3%	87.5%	83.2%
early washout	0.632	46.9%	79.6%	51.1%	76.8%

AUC, Area under the ROC curve; Se, sensitivity; Sp, specificity; PPV, positive predictive value and NPV, negative predictive value.

### 3.3 Defining CEUS high-risk

Given these results, we considered in the next step that the CEUS characteristics predictive of malignancy are inhomogeneous enhancement, the presence of enhancement defects and complete hypoenhancement. If any of these was present, the nodule was considered as **high-risk in CEUS**.

**Figure 3:**
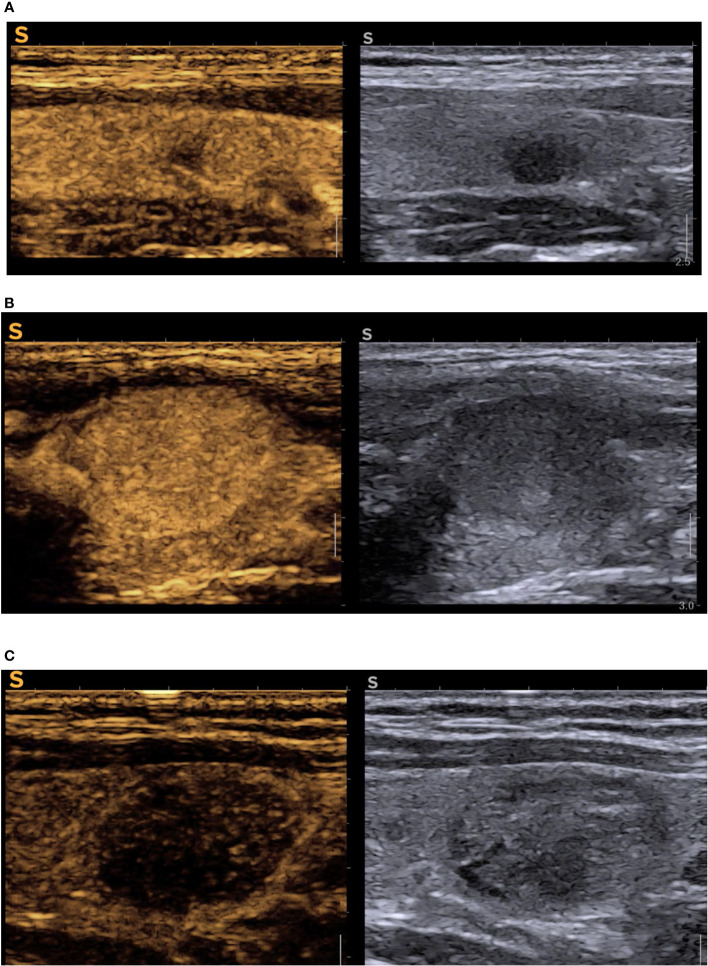
CEUS cases **(A)** oval, intense hypoechoic nodule with ill-defined nodules in B-mode (EU-TIRADS 5), right side of the image, reconfirmed high-risk in CEUS with inhomogeneous hypoenhancement, central enhancement defect, absent peripheral ring, in left half of the image = PTC; **(B)** hypoechoic subcapsular solid nodule (EU-TIRADS 4) with peripheral enhanced ring, complete iso-enhancement, no enhancement defects in CEUS (remained TIRADS 4, but it was low-risk in CEUS) = benign nodule; **(C)** solid, well-defined, isoechoic with some areas of hypoechogenicity which included the nodule in EU TIRADS 4, CEUS high risk due to diffuse hypoechancement (upgraded to modified-TIRADS 5) = medullary thyroid cancer. CEUS, contrast-enhanced ultrasound; TIRADS, Thyroid Imaging Reporting and Data System.

**Figure 3** describes the high-risk feature of malignancy in CEUS evaluation.

**Figure 4:**
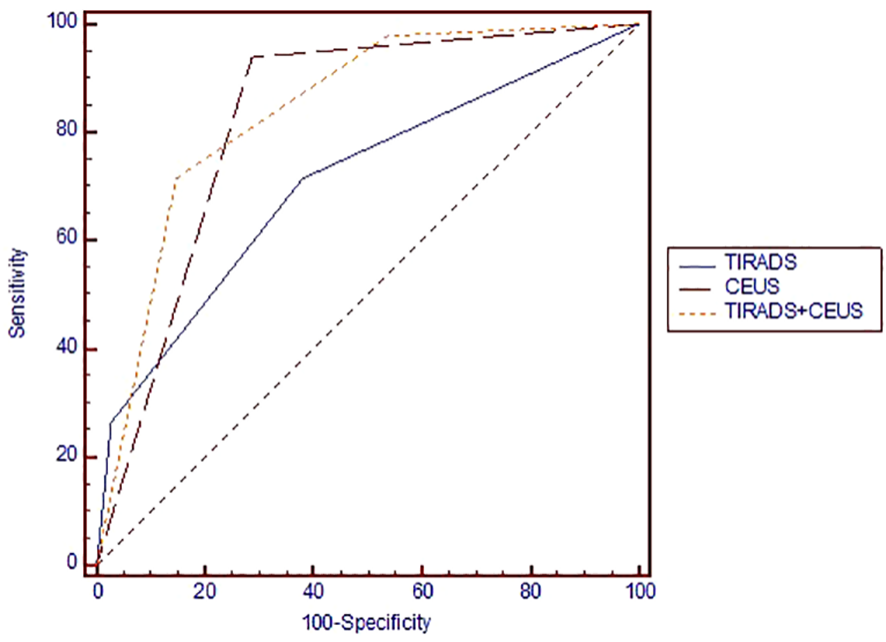
Area under the ROC curve for comparing the diagnostic performance of TIRADS, CEUS and TIRADS+CEUS scores in evaluating the risk of thyroid cancer. ROC, receiver operating curve; TIRADS, Thyroid Imaging Reporting and Data System; CEUS, contrast-enhanced ultrasound.

We obtained an AUC of 0.825 (**Figure 4**) for thyroid CEUS evaluation, which indicates good diagnostic accuracy. The high sensitivity of 93.9% means it’s very effective at identifying true positive cases of thyroid cancer, while the specificity of 71.3% shows it is moderately accurate at ruling out those without cancer.

### 3.4 Combined TIRADS + CEUS

The AUC of 0.840 for TIRADS combined with CEUS indicates high diagnostic accuracy. We considered TIRADS scores 4 and 5 as predictive for cancer, the obtained sensitivity was high at 97.96%, but specificity is low 46.3%. The PPV is 45.3%, and NPV is at 98.0%. If high-risk would have been considered only category 5 in this classification, we would obtain a better overall diagnostic performance, given the balance between sensitivity (71.43%) and specificity (85.19%), and PPV 68.6%, NPV 86.8%, but some cancers would be missed.

The comparative diagnostic effectiveness of TIRADS, CEUS, and a combined TIRADS+CEUS approach in assessing thyroid cancer risk is illustrated in **Figure 3** through a Receiver Operating Characteristic (ROC) curve.

For evaluating their efficacy in thyroid cancer risk assessment, **Table 4** provides detailed values of the diagnostic performance of each method in terms of AUC parameters and **Table 5** – in terms of likelihood intervals.

**Table 4:** Compared diagnostic performance for the evaluated algorithms: TIRADS, CEUS and TIRADS + CEUS.

	AUC	Se	Sp	PPV	NPV
**TIRADS**	0.707	71.4%	62%	46.1%	82.7%
**CEUS**	0.825	93.9%	71.3%	59.7%	96.2%
**TIRADS+CEUS**	0.840	97.96%	46.3%	45.3%	98%

**Table 5:** Interval likelihood ratios for TIRADS and TIRADS+CEUS.

SCORE	Positive	Negative	Likelihood ratio	95% CI
TIRADS				
**3**	14	67	0.461	0.289 to 0.734
**4**	22	38	1.276	0.854 to 1.908
**5**	13	3	9.551	2.850 to 32.002
TIRADS + CEUS				
**3**	1	50	0.0441	0.00627 to 0.310
**4**	13	42	0.682	0.405 to 1.150
**5**	35	16	4.821	2.967 to 7.836
**Total**	49	108		

In our analysis of diagnostic methods for thyroid cancer, we observed distinct variations in the post-test probabilities between TIRADS, CEUS, and the combined TIRADS+CEUS approach. The Positive Post-Test Probability (PPT) for TIRADS alone was 46.02%, indicating a moderate likelihood of accurately confirming thyroid cancer. However, its Negative Post-Test Probability (NPT) was 19.64%, suggesting a limitation in its ability to reliably exclude the disease. In contrast, CEUS alone showed a higher PPT of 59.75%, reflecting greater accuracy in confirming cancer presence, and a lower NPT of 29.32%, indicating improved reliability in ruling out cancer. The most noteworthy findings emerged from the combined TIRADS+CEUS approach, which yielded a PPT of 68.64% and an NPT of 23.16%. These results underscore the enhanced diagnostic accuracy achieved when integrating both methods, with a notably higher probability of correctly identifying thyroid cancer cases and a more balanced approach in excluding the disease compared to TIRADS or CEUS used independently.

## 4 Discussion

There are significant similarities in the characteristics of benign and malignant features in the conventional US examination. For example, carcinomas can also exhibit a halo sign, hypoechogenicity is sometimes present in adenomas or in nodular autoimmune disease, and microcalcifications are overly diagnosed (26, 27). Moreover, it was demonstrated that EU-TIRADS-defined suspicious ultrasonographic features are less common in follicular thyroid carcinoma (FTC) than in papillary (PTC) and medullary thyroid carcinomas (MTC). Consequently, the FNA indication rate based on TIRADS assessment for FTC was observed to be notably lower at 55.5%, compared to 85.0% for PTC and 88.9% for MTC. This significant discrepancy highlights the need for adjustments in EU-TIRADS to better detect FTC early (28). Regarding FNA, approximately 50% of all biopsied nodules are confirmed to be benign, whereas more than 25% show unclear cytological findings (29).

Given these, thyroidologists are currently exploring additional imaging features to enhance the accuracy of diagnosis, such as elastography and more recently CEUS, with contrast administration linked to an adverse event rate that is nearly negligible (1:10,000) (29, 30). Current data on the effectiveness of CEUS in evaluating thyroid nodules is heterogeneous (31–33). At this stage, further research from various regions and conducted by skilled operators is necessary to formulate conclusive and reliable recommendations. Our study adds CEUS to the European TIRADS.

In our analysis, certain CEUS parameters demonstrated exceptional accuracy in detecting cancer. The parameter with the highest AUC, indicating the best overall diagnostic accuracy, was the complete, hyperenhanced peripheral ring. The presence of a complete, peripheral ring was described in one cancer case, a classic papillary carcinoma. Our results are in concordance with the published data, with good benignity predictions for the presence of this feature: sensitivity of 97.6%, specificity of 98.7%, and accuracy of 98.3% (25). Furthermore, it has been reported that the presence of an irregular peripheral ring is suggestive of malignancy (25). The regular, hyperenhanced peripheral ring is thought to be linked to compression on the capsule and peripheral parenchymal vessels surrounding the nodule, similar to the appearance of a halo sign observed in conventional ultrasound (24). An inhomogeneous enhancement showed good diagnostic accuracy in our study, with an AUC of 0.818 and high sensitivity. However, its specificity and PPV are relatively lower, which may lead to a higher rate of false positives. Enhancement defects also had a notably high specificity and PPV. The parameters hypoenhancement and early-washout had lower AUC values (0.695 and 0.632, respectively), suggesting less diagnostic accuracy compared to other parameters. Their lower sensitivity values also imply a higher chance of missing thyroid cancers.

Considering our results, thyroid CEUS is a reliable tool for diagnosis, with a strong ability to detect the condition but with a moderate rate of false positives. The overall diagnostic performance of CEUS was described to be good. A recent meta-analysis (29) conducted using histology reference found combined sensitivity and specificity of 85% and 82%, respectively, with consistent sensitivities, and slight inconsistencies for specificity. Other authors have also reported comparable findings, reporting to FNA alone (22, 23). The overall accuracy described in our study was in line with these results - 78.3%, with 93.9% sensitivity and 71.3% specificity.

In our TIRADS+CEUS approach, assigning scores of 4 and 5 as indicators of cancer presented a high sensitivity of 97.96% but a lower specificity of 46.3%. Considering only score 5 as high-risk would improve specificity (85.19%), but our primary aim was to detect as many cancers as possible, hence our choice to keep both scores 4 and 5 as high-risk. Our algorithms demonstrate that incorporating Contrast-Enhanced Ultrasound (CEUS) notably enhances specificity, particularly for nodules initially classified as category 4 in TIRADS; thus, the application of CEUS in this category is where it could yield the most significant benefit. There were other proposals of CEUS + TIRADS approach, with slightly different designs but also with improved outcomes One study found that when used together in a predictive model, TIRADS and CEUS they performed better than either method alone (P <0.05), with an accuracy of 86.6% for the combined score (21). In another multicentric study on a large number of cases, the authors found au AUC for the modified TIRADS of 0.936, sensitivity 93.6%, specificity 88.5%, with Kappa for CEUS evaluation of 0.81, describing good interobserver variability (34). In our group, for the B-mode-only TIRADS, the percentage of cancers identified in score 3 is 17.28%, in score 4 it is 57.9% and 81.25% in score 5; for the TIRADS + CEUS, in score 3 we identified 1.9% cancers, in score 4 we identified 23.6% and in score 5 – 68%. Cancer was missed in only one case in TIRADS + CEUS score 3, but there were 42 false positive cases in score 4 and 16 false positives in score 5. The low sensitivity of hypoenhancement in CEUS for detecting thyroid cancer presents notable limitations, primarily due to variability in nodule vascularity and the subjective nature of interpreting enhancement defects, operators must take into consideration that looking for this criteria only might result in failing to identify some cancers.

Considering our findings, it’s evident that integrating TIRADS with CEUS significantly enhances the diagnostic accuracy for thyroid cancer. The combined approach shows a notable improvement in both confirming and ruling out the disease, as reflected by higher positive and more balanced negative post-test probabilities, compared to the individual use of TIRADS or CEUS. This synergy suggests a more reliable diagnostic strategy, particularly in unclear cases. However, it is important to mention that these numbers may not applicable in the general population because the prevalence of cancer in the study population is higher due to selection of nodules with surgical indication for study inclusion.

## 5 Conclusions

The integration of TIRADS with CEUS could become a cornerstone in diagnostic ultrasound imaging for thyroid cancer, especially in ambiguous cases. This approach enhances both the confirmation and exclusion of the disease, leading to more reliable diagnostic outcomes. Key CEUS parameters such as the ‘complete, hyperenhanced peripheral ring’ are predictive of benignity, while ‘inhomogeneous enhancement’, ‘enhancement defects’, and ‘complete hypoenhancement’ are indicative of thyroid cancer.

## Data availability statement

The raw data supporting the conclusions of this article will be made available by the authors, without undue reservation.

## Ethics statement

The studies involving humans were approved by Spitalul Judetean De Urgenta Pius Brinzeu Timisoara. The studies were conducted in accordance with the local legislation and institutional requirements. The participants provided their written informed consent to participate in this study.

## Author contributions

AB: Writing – review & editing, Writing – original draft, Software, Resources, Project administration, Methodology, Investigation, Funding acquisition, Conceptualization. LM: Writing – review & editing, Visualization, Validation, Methodology, Investigation, Formal analysis, Data curation. AP: Writing – review & editing, Visualization, Validation, Supervision, Resources. FB: Writing – review & editing, Supervision, Resources, Project administration, Investigation. DS: Writing – review & editing, Writing – original draft, Visualization, Supervision, Resources, Project administration, Methodology, Investigation.
